# Insertion/Deletion Polymorphisms in the Promoter Region of *BRM* Contribute to Risk of Hepatocellular Carcinoma in Chinese Populations

**DOI:** 10.1371/journal.pone.0055169

**Published:** 2013-01-24

**Authors:** Xueren Gao, Moli Huang, Limin Liu, Yan He, Qiang Yu, Hua Zhao, Chunxiao Zhou, Jinkun Zhang, Zhansheng Zhu, Jiao Wan, Xinghong Jiang, Yuzhen Gao

**Affiliations:** 1 Department of Forensic Medicine, Medical College of Soochow University, Suzhou, Jiangsu, People’s Republic of China; 2 Department of Bioinformatics, Medical College of Soochow University, Suzhou, Jiangsu, People’s Republic of China; 3 Department of Pathophysiology, Medical College of Soochow University, Suzhou, Jiangsu, People’s Republic of China; 4 Department of Gastroenterology, Affiliated Hospital of Nanjing Medical University, Suzhou, Jiangsu, People’s Republic of China; 5 Department of General Surgery, The First Affiliated Hospital of Soochow University, Suzhou, Jiangsu, People’s Republic of China; 6 Department of Neurobiology and Psychology, Key Laboratory of Pain Research & Therapy, Medical College of Soochow University, Suzhou, Jiangsu, People’s Republic of China; The University of Texas M. D. Anderson Cancer Center, United States of America

## Abstract

**Background:**

BRM (Brahma homologue) is well known for its critical role in tumor suppression and cancer development. Genetic variations in the promoter region of *BRM* have been suggested to be associated with loss of BRM expression and lung cancer risk. To the authors’ knowledge, no study on the role of *BRM* genetic polymorphisms in hepatocellular carcinoma (HCC) risk has been performed.

**Methodology/Principal Findings:**

In two independent case-control studies containing 796 HCC cases and 806 cancer-free individuals, we genotyped two putative functional insertion/deletion (indel) polymorphisms [BRM-1321 (rs3832613) and BRM-741 (rs34480940)] within promoter region of *BRM* in Chinese populations using a PCR-based method. Real-time RT-PCR analysis was used to explore the genotype-phenotype correlation between these polymorphisms and BRM expression in both tissue samples and HCC cell lines. Logistic regression analysis showed that compared to BRM-1321del/del genotype, the ins/del and ins/ins variant genotypes had an increased HCC risk [adjusted odds ratio (OR) = 1.47, 95% confidence interval (CI) = 1.19–1.82; adjusted OR = 2.55, 95% CI = 1.75–3.72, respectively]. No significant association between BRM-741 and HCC incidence was observed. However, stratification analysis revealed a significant association between ins/ins genotype of BRM-741 and increased HCC susceptibility in smokers (adjusted OR = 2.07, 95% CI = 1.33–3.22). Quantitative PCR analyses demonstrated that the genotypes of BRM-1321 and the corresponding haplotypes were significantly correlated with BRM expression *in vivo*. Compared with ins/ins genotype, subjects carrying ins/del and del/del genotype had 2.30 and 4.99 fold higher BRM expression in HCC tissue samples, respectively. Similar trends were observed in western blot analysis at protein level.

**Conclusions/Significance:**

Our findings suggest that *BRM* promoter polymorphism (BRM-1321) could regulate BRM expression and may serve as a potential marker for genetic susceptibility to HCC.

## Introduction

Hepatocellular carcinoma (HCC) is the most common primary malignancy of liver and its mortality rate is the third highest among the most common cancers [1]. Over 80% of HCC cases are from the Asian and African continents, and more than 50% of cases are from mainland China [2]. Epidemiological and clinical studies have demonstrated that the major risk factors for HCC include alcoholism, hepatitis B virus (HBV) and hepatitis C (HCV), aflatoxin, liver cirrhosis [3], [4]. As the important carcinogen for HCC, HBV infection has become a significant public health problem in China [5]. Accumulated evidences from molecular genetics indicate that individual’s genetic and epigenetic factors are involved in their susceptibility to HCC [3]. Recent genome wide association studies (GWAS) have also identified several new susceptibility loci for HCC [6], which is helpful to predict individual and population risk and clarify pathophysiologic mechanisms relevant to HCC. However, to date, the molecular carcinogenic mechanism of HCC is still not fully elucidated.

The SWI/SNF (Switch/sucrose non-fermentable) complexes mediate chromatin remodeling processes in an ATP-dependent manner that is essential for gene expression, cell cycle control, differentiation, proliferation and DNA repair [7]. The mammalian complexes are comprised of a highly related family of multi-subunit complexes and play critical roles in tumor suppression [8]. Emerging evidence indicates that BRM (Brahma homologue), a key SWI/SNF complex subunit, is silenced in 15–20% of various solid tumors [9]. Recurrent mutations in subunits of the complex have been identified in many cancers including lung cancer and breast cancer, providing a novel link between chromatin remodeling and tumorgenesis [10], [11]. In addition, BRM has been found preferentially expressed in human liver [12]. Therefore, there is such possibility that the genetic polymorphisms in these subunit genes and their interactions with environmental factors may alter the susceptibility to HCC. However, there are no related studies concerning the association between *BRM* genetic variations and HCC incidence.

Recently, two insertion/deletion (indel) polymorphisms have newly identified in the promoter region (−1321 bp and −741 bp upstream of the BRM transcription start site, i.e. rs3832613 and rs34480940) of BRM and have been proved to be associated with loss of BRM expression and lung cancer risk [13]. Moreover, *in-silico* analysis has also revealed that these indels are located within the binding site of putative transcription factor (i.e. myocyte enhancer factor-2) [13]. Thus, we hypothesized that these novel indel variations in the promoter region of *BRM* were associated with altered BRM expression and HCC risk. In the current study, we conducted two independent case-control studies in Chinese populations to investigate the associations between these two indel polymorphisms and HCC risk. Consecutive functional assays were used to assess the possible functional significance of these polymorphisms.

## Materials and Methods

### Ethics Statement

This study was approved by the Ethical Committee of Soochow University. Written informed consent was obtained from each participant before investigation.

### Study Populations

Our study included two independent case-control sets containing 796 newly diagnosed incident HCC cases and 806 cancer-free controls who were genetically unrelated ethnic Han Chinese. For the first case control set (panel I), 408 HCC patients were recruited from May 2007 to July 2010 at the affiliated hospitals of Soochow University. In the second case control set (panel II), 388 HCC patients were recruited from March 2005 to October 2010 at the affiliated hospital of Nanjing Medical University. None of these HCC patients had received any medical treatment. The diagnosis of the cases, the inclusion and exclusion criteria for the cases and controls, and the classification of smoking and drinking status were previously described [14]–[16]. Controls with frequency-matched age (±5 years) and sex were cancer-free individuals selected from a community nutritional survey that was conducted in the same regions during the same period as recruitment of HCC patients. Genomic DNA was extracted from the peripheral blood of cases and controls. Tumor stages were determined according to a modified American Joint Committee on Cancer (AJCC) and international union against cancer (UICC) standard. Each subject was interviewed in-person using a structured questionnaire to obtain information on demographic data and related risk factors, including smoking and drinking status. All participants were negative for antibodies to hepatitis C virus, hepatitis D virus or HIV.

For functional assay, additional 72 tumor tissues and adjacent non-tumor tissues from patients with a diagnosis of HCC were collected according to the availability of frozen stored tissue from HCC resections from June 2004 to May 2006 at Department of General Surgery, the First Affiliated Hospital of Soochow University. All cases had histological confirmation of their tumor diagnosis and none of these patients had received any preoperative chemotherapy or radiotherapy. After surgical resection, the fresh tissues were immediately stored at −80°C until the DNA/RNA/protein extraction for the current study.

### DNA Extraction and Genotyping

Genomic DNA of blood samples, tissues and hepatoma cell lines were isolated using genomic DNA purification kit (Qiagen). DNA fragments containing rs34480940 and rs3832613 were amplified with two pairs of genotyping primers (BRM-741-F: 5′-TTGTGCCCGCCTCCCTTTTC-3′, BRM-741-R: 5′-GGCTCCGAGTGGCACCAAAG-3′, BRM-1321-F: 5′-GGGAAGAATCCTCAACCAGATAGTC-3′, BRM-1321-R: 5′-GTTTTATGAAGTGTGAAAGAATGTTAGG-3′), respectively. The PCR products were analyzed by 7% non-denaturing polyacrylamide gel electrophoresis and visualized by silver staining [17]. The genotypes were determined by the numbers and the length of the band(s) in the gels. To validate the genotyping method, we also analyzed 50 randomly selected DNA samples by direct sequencing. The coincidence rate of these two methods was 100%, suggesting that the PCR-based method was reliable. Approximately 10% of the samples were randomly selected and examined in duplicates by independent researchers, and the reproducibility was 100%.

### Real-time RT-PCR Analysis

The Hep3B, Huh-7, sk-Hep-1 and SMMC-7721 hepatoma cell lines were obtained directly from Shanghai Cell Bank of Chinese Academy of Sciences and cultured in Dulbecco’s Modified Eagle’s Medium supplemented with 10% fetal bovine serum (FBS) and 1% penicillin-streptomycin at 37°C in a humidified 5% CO_2_ incubator. The cell lines were characterized at the bank using short tandem repeat (STR)-based fingerprinting analysis except SMMC-7721. All cell lines were used within three months of thawing fresh vials. Total RNA was isolated from tissue specimens and cell lines using RNA isolation kit of Qiagen and then converted to cDNA using random primers and Superscript II (Invitrogen). A SYBR® Green gene expression assay was performed using Roche LightCycler® 480 to quantify relative BRM expression in these samples. GAPDH was chosen as the internal control. Primer sequences used for BRM and GAPDH were as follow: BRM-F: 5′-GATTGTAGAAGACATCCATTGTGG-3′, BRM-R: 5′-GACATATAACCTTGGCTGTGTTGA-3′, GAPDH-F: 5′-CTCTCTGCTCCTCCTGTTCGAC-3′, GAPDH-R: 5′-TGAGCGATGTGGCTCGGCT-3′. Negative controls consisted of distilled H_2_O. The expression levels of BRM were normalized with GAPDH using a 2^−ΔΔCT^ method. In all cases, the relative lower BRM expression group was used as calibrator (fold change  = 1). A melting curve analysis was performed for the PCR products to evaluate primer specificity.

### Western Blot

To further investigate the correlation between BRM-1321 genotype and BRM protein level, 3 randomly selected HCC tumor tissues and adjacent non-tumor tissues with different genotypes or haplotypes were analyzed by western blot. Approximately 40 µg of protein extract from tissues samples were separated on 8% polyacrylamide gel. Proteins were transferred to a PVDF membrane (GE Healthcare) and probed with primary antibodies against BRM (1∶500, Santa Cruz Biotechnology) and GAPDH (1∶1000, Santa Cruz Biotechnology). The primary antibodies were detected by horseradish peroxidase (HRP)-conjugated secondary antibodies (1∶1000, Santa Cruz Biotechnology). Films were exposed in dark room using an enhanced chemiluminescence system (ECL, Cell Signaling Technologies).

### Statistical Analysis

The Hardy-Weinberg equilibrium was analyzed using χ^2^ test. Unconditional logistic regression was used to assess the associations between the indel polymorphisms and HCC risk, adjusted by sex, age, smoking, drinking and HBV infection status. In the stratification analysis, we assessed the main effect of the indel polymorphisms in each subgroup and the possible interaction between polymorphisms and selected variables on cancer risk. A multiplicative interaction was suggested when OR11> OR10 × OR01, in which OR11 is the OR when both factors were present, OR01 is the OR when only factor 1 was present and OR10 is the OR when only factor 2 was present to evaluate the possible gene-environment interactions on HCC risk. The *P* values of test for the multiplicative interaction between the two indel polymorphisms and selected variables on cancer risk were calculated using unconditional logistic regression model. Due to relative small sample size, former smoker and current smoker, light drinker and heavy drinker were integrated into one group in stratification analysis, respectively. Haplotype frequencies as well as linkage disequilibrium (LD) were estimated from genotype data using the SHESIS program with default parameters [18]. The normalized expression values of BRM in HCC tumor tissue and adjacent non-tumor tissue samples were compared using the paired *t* test. The normalized expression levels of BRM among different genotype or haplotype groups were compared using one way ANOVA. These statistical analyses were implemented in Statistic Analysis System software (version 8.0, SAS Institute). *P*<0.05 was used as the criterion of statistical significance, and all statistical tests were two sided.

## Results

### The Associations of BRM Indel Polymorphisms with HCC Susceptibility

**Figure 1:**
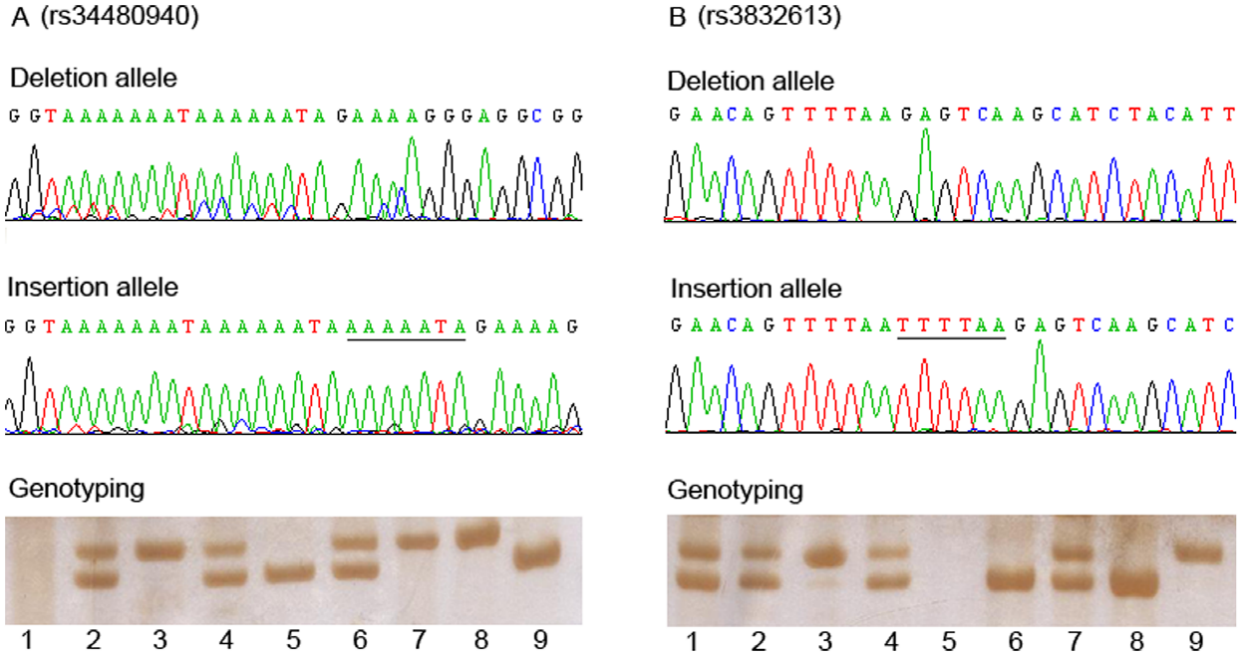
Example sequencing and genotyping output for the two BRM Indel polymorphisms. The upper and middle panels showed the sequence of deletion and insertion allele, respectively. The underlined base-pairs indicate the inserted sequences. The lower panel displays an example of the genotyping assay results. For rs34480940 (Figure 1A), lane 5 and 9, del/del genotype; lane 2, 4 and 6, ins/del genotype; lane 1, negative control; remaining lanes, ins/ins genotype. For rs3832613 (Figure 1B), lane 3 and 9, ins/ins genotype; lane 3 and 9, del/del genotype; lane 5, negative control; remaining lanes, ins/del genotype.

The demographic characteristics of the 796 HCC patients and 806 controls from two independent case-control sets were summarized in Table 1. There were no statistically significant differences in terms of the frequency distribution of sex, age, smoking and drinking status, suggesting that the frequency matching was adequate. Approximately 71.4% of the cases and 13.4% of the controls were HBsAg-positive, in accordance with the fact that HBV infection was a major risk factor for HCC. Example output from sequencing and genotyping assays of two polymorphisms were shown in Figure 1. The observed genotype frequencies for the two indel polymorphisms were consistent with those expected from the Hardy-Weinberg equilibrium in both cases and controls (all *P* values >0.05).

**Table 1:** Demographic characteristics among HCC cases and controls.

	Overall			Panel I			Panel II		
Characteristics	Case (n = 796)	Control (n = 806)	*P*	Case (n = 408)	Control (n = 408)	*P*	Case (n = 388)	Control (n = 398)	*P*
Age(mean±SD)	51.9±11.8	51.5±11.7	0.38^a^	53.0±12.5	52.1±11.9	0.30^a^	49.2±10.7	50.5±11.5	0.42^a^
Gender, N (%)									
Male	529(66.5)	535(66.4)	0.97^b^	274(67.2)	272(66.7)	0.88^b^	255(65.7)	263(66.1)	0.92^b^
Female	267(33.5)	271(33.6)		134(32.8)	136(33.3)		133(34.3)	135(33.9)	
Smoking Status									
Nonsmokers	462(58.0)	455(56.5)	0.81^b^	241(59.1)	237(58.1)	0.95^b^	221(57.0)	218(54.8)	0.80^b^
Former Smokers	170(21.4)	180(22.3)		85(20.8)	86(21.1)		85(21.9)	94(23.6)	
Current smoker	164(20.6)	171(21.2)		82(20.1)	85(20.8)		82(21.1)	86(21.6)	
Drinking status									
Nondrinker	412(51.8)	429(53.2)	0.84^b^	211(51.7)	214(52.5)	0.97^b^	201(51.8)	215(54.0)	0.80^b^
Light Drinker	291(36.6)	285(35.4)		152(37.3)	151(37.0)		139(35.8)	134(33.7)	
Heavy Drinker	93(11.7)	92(11.4)		45(11.0)	43(10.5)		48(12.4)	49(12.3)	
Tumor stages									
Ia+Ib	552(69.3)			289(70.8)			263(67.8)		
IIa+IIb	173(21.7)			86(21.1)			87(22.4)		
IIIa+IIIb	71(8.9)			33(8.1)			38(9.8)		
HBsAg, N (%)									
Positive	568(71.4)	108(13.4)	<0.0001^b^	294(72.1)	61(15.0)	<0.0001^b^	274(70.6)	47(11.8)	<0.0001^b^
Negative	228(28.6)	698(86.6)		114(27.9)	347(85.0)		114(29.4)	351(88.2)	

a Two-sided two-sample *t*-test between cases and controls.

b χ^2^ test for differences between cases and controls.

Genotype frequencies and odds ratio (OR) and 95% confidence interval (CI) for cases and controls are presented in Table 2. Under co-dominant model, compared with the del/del wild genotype, subjects with the heterozygous ins/del or homozygous ins/ins variants of BRM-1321 had a significantly increased risk of HCC in a dose dependent manner (adjusted OR = 1.47, 95% CI = 1.19–1.82; adjusted OR = 2.55, 95% CI = 1.75–3.72, respectively). Similar trends were observed in both panels. Each additional copy of the insertion allele was associated with a 55% increased risk in pooled analysis (OR = 1.55, 95% CI = 1.33–1.81, *P*<0.0001). However, logistic regression analysis revealed that there was no significant association between BRM-741 and HCC in both panels. Only a borderline significant association was observed in the pooled analysis for ins/ins genotype (*P* = 0.05).

**Table 2:** Associations between *BRM* promoter Indel genotypes and HCC risk.

Variations	Population	Genotype	Cases	%	Control	%	OR (95% CI)^a^	*P*
BRM-1321	Panel I	del/del	170	41.7	220	53.9	1.00(reference)	
		ins/del	182	44.6	160	39.2	1.47(1.09–2.00)	0.009
		ins/ins	56	13.7	28	6.9	2.58(1.53–4.36)	0.0001
	*P*_trend_						<0.0001	
	Panel II	del/del	163	42.0	215	54.0	1.00(reference)	
		ins/del	177	45.6	158	39.7	1.47(1.08–2.00)	0.01
		ins/ins	48	12.4	25	6.3	2.52(1.45–4.41)	0.0004
	*P*_trend_						0.0001	
	Overall	del/del	333	41.8	435	54.0	1.00(reference)	
		ins/del	359	45.1	318	39.5	1.47(1.19–1.82)	0.0002
		ins/ins	104	13.1	53	6.6	2.55(1.75–3.72)	<0.0001
	*P*_trend_						<0.0001	
BRM-741								
	Panel I	del/del	83	20.3	94	23.0	1.00(reference)	
		ins/del	188	46.1	194	47.5	1.09(0.75–1.58)	0.61
		ins/ins	137	33.6	120	29.4	1.28(0.85–1.91)	0.19
	*P*_trend_						0.18	
	Panel II	del/del	75	19.3	90	22.6	1.00(reference)	
		ins/del	184	47.4	192	48.2	1.16(0.79–1.70)	0.46
		ins/ins	129	33.2	116	29.1	1.35(0.89–2.04)	0.15
	*P*_trend_						0.15	
	Overall	del/del	158	19.8	184	22.8	1.00(reference)	
		ins/del	372	46.7	386	47.9	1.12(0.86–1.46)	0.38
		ins/ins	266	33.4	236	29.3	1.31(0.98–1.74)	0.05
	*P*_trend_						0.05	

a adjusted for sex, age, smoking status, drinking status and HBV infection.

Furthermore, we performed stratified analyses by smoking status, drinking status and HBV infection for BRM-741 and BRM-1321 indel polymorphisms. Because of low number of HBsAg-positive subjects in control group and HBsAg-negative subjects in case group, we analyzed the pooled data from two case control sets. As shown in Table 3, Table 4 and Table 5, these common confounders did not seem to affect the positive association between BRM-1321 and risk of HCC (all *P*_interaction_ values >0.05). Intriguingly, we observed a significant association between ins/ins genotype of BRM-741 and HCC incidence in smokers subgroup (Table 3) (*P*_interaction_ = 0.02). No significant association was observed in non-smoker subgroup. Other parameters did not contribute to the association between BRM-741 and HCC risk (Table 3, Table 4 and Table 5).

**Table 3:** Stratification analysis based on smoking status in two populations.

Variations	Population	Genotype	Smokers			Nonsmokers			*P*_interaction_
			Case, %	Control, %	OR(95% CI)^a^	Case, %	Control, %	OR(95% CI)^a^	
BRM-1321	Panel I	del/del	70(41.9)	90(52.6)	1.00(Reference)	100(41.5)	130(54.8)	1.00(Reference)	0.69
		ins/del	73(43.7)	66(38.6)	1.49(0.91–2.42)	109(45.2)	94(39.7)	1.48(0.99–2.20)	
		ins/ins	24(14.4)	15(8.8)	**2.30(1.05–5.10)**	32(13.3)	13(5.5)	**3.10(1.47–6.62)**	
	*P*_trend_				0.03			0.0004	
	Panel II	del/del	70(41.9)	99(55.0)	1.00(Reference)	93(42.1)	116(53.2)	1.00(Reference)	0.87
		ins/del	74(44.3)	70(38.9)	1.50(0.93–2.41)	103(46.6)	88(40.4)	1.46(0.97–2.22)	
		ins/ins	23(13.8)	11(6.1)	**2.67(1.14–6.36)**	25(11.3)	14(6.4)	**2.90(1.28–6.65)**	
	*P*_trend_				0.003			0.009	
	Overall	del/del	140(41.9)	189(53.8)	1.00(Reference)	193(41.8)	246(54.1)	1.00(Reference)	0.97
		ins/del	147(44.0)	136(38.7)	**1.49(1.07–2.09)**	212(45.9)	182(40.0)	**1.47(1.11–1.96)**	
		ins/ins	47(14.1)	26(7.4)	**2.38(1.35–4.18)**	57(12.3)	27(5.9)	**3.01(1.75–5.19)**	
	*P*_trend_				0.0003			<0.0001	
BRM-741	Panel I	del/del	30(18.0)	44(25.7)	1.00(Reference)	53(22.0)	50(21.1)	1.00(Reference)	0.13
		ins/del	71(42.5)	79(46.2)	1.34(0.73–2.44)	117(48.5)	115(48.5)	0.97(0.59–1.58)	
		ins/ins	66(39.5)	48(28.1)	**2.06(1.09–3.91)**	71(29.5)	72(30.4)	0.92(0.53–1.57)	
	*P*_trend_				0.02			0.78	
	Panel II	del/del	30(18.0)	47(26.1)	1.00(Reference)	45(20.4)	43(19.7)	1.00(Reference)	0.16
		ins/del	75(44.9)	85(47.2)	1.36(0.76–2.47)	109(49.3)	107(49.1)	0.98(0.58–1.66)	
		ins/ins	62(37.1)	48(26.7)	**2.07(1.09–3.92)**	67(30.3)	68(31.2)	0.96(0.54–1.70)	
	*P*_trend_				0.02			0.82	
	Overall	del/del	60(18.0)	91(25.9)	1.00(Reference)	98(21.2)	93(20.4)	1.00(Reference)	0.02
		ins/del	146(43.7)	164(46.7)	1.35(0.89–2.04)	226(48.9)	222(48.8)	0.97(0.68–1.39)	
		ins/ins	128(38.3)	96(27.4)	**2.07(1.33–3.22)**	138(29.9)	140(30.8)	0.94(0.64–1.38)	
	*P*_trend_				0.0007			0.72	

a adjusted by age, sex, drinking status and HBV infection status. Smokers included former smokers and current smokers.

**Table 4:** Stratification analysis based on drinking status in two populations.

Variations	Population	Genotype	Drinkers			Nondrinkers			*P*_interaction_
			Case, %	Control, %	OR(95% CI)^a^	Case, %	Control, %	OR(95% CI)^a^	
BRM-1321	Panel I	del/del	80(40.6)	105(54.1)	1.00(Reference)	90(42.7)	115(53.7)	1.00(Reference)	0.96
		ins/del	87(44.2)	74(38.1)	1.53(0.98–2.39)	95(45.0)	86(40.2)	1.40(0.92–2.13)	
		ins/ins	30(15.2)	15(7.7)	**2.51(1.20–5.30)**	26(12.3)	13(6.1)	**2.44(1.12–5.36)**	
	*P*_trend_				0.002			0.006	
	Panel II	del/del	78(41.7)	100(54.6)	1.00(Reference)	85(42.3)	115(53.5)	1.00(Reference)	0.94
		ins/del	85(45.5)	72(39.3)	1.50(0.95–2.36)	92(45.8)	86(40.0)	1.43(0.94–2.20)	
		ins/ins	24(12.8)	11(6.0)	**2.77(1.21–6.45)**	24(11.9)	14(6.5)	**2.30(1.06–5.01)**	
	*P*_trend_				0.004			0.009	
	Overall	del/del	158(41.1)	205(54.4)	1.00(Reference)	175(42.5)	230(53.6)	1.00(Reference)	0.93
		ins/del	172(44.8)	146(38.7)	**1.51(1.11–2.07)**	187(45.4)	172(40.1)	**1.42(1.05–1.90)**	
		ins/ins	54(14.1)	26(6.9)	**2.62(1.52–4.52)**	50(12.1)	27(6.3)	**2.36(1.38–4.06)**	
	*P*_trend_				<0.0001			0.0002	
BRM-741	Panel I	del/del	39(19.8)	45(23.2)	1.00(Reference)	44(20.9)	49(22.9)	1.00(Reference)	0.97
		ins/del	90(45.7)	91(46.9)	1.12(0.64–1.94)	98(46.4)	103(48.1)	1.07(0.63–1.80)	
		ins/ins	68(34.5)	58(29.9)	1.30(0.72–2.36)	69(32.7)	62(29.0)	1.22(0.69–2.16)	
	*P*_trend_				0.27			0.41	
	Panel II	del/del	38(20.3)	40(21.9)	1.00(Reference)	37(18.4)	50(23.3)	1.00(Reference)	0.82
		ins/del	85(45.5)	88(48.1)	1.03(0.58–1.82)	99(49.3)	104(48.4)	1.30(0.76–2.23)	
		ins/ins	64(34.2)	55(30.1)	1.21(0.65–2.23)	65(32.3)	61(28.4)	1.42(0.79–2.56)	
	*P*_trend_				0.45			0.21	
	Overall	del/del	77(20.1)	85(22.5)	1.00(Reference)	81(19.7)	99(23.1)	1.00(Reference)	0.96
		ins/del	175(45.6)	179(47.5)	1.07(0.73–1.58)	197(47.8)	207(48.3)	1.17(0.81–1.70)	
		ins/ins	132(34.4)	113(30.0)	1.26(0.83–1.91)	134(32.5)	123(28.7)	1.31(0.88–1.96)	
	*P*_trend_				0.19			0.14	

a adjusted by age, sex, smoking status and HBV infection status. Drinkers included light drinkers and heavy drinkers.

**Table 5:** Stratification analysis based on HBV infection status.

Variations	Genotype	HBV Positive			HBV Negative			*P*_interaction_
		Case, %	Control, %	OR(95% CI)^a^	Case, %	Control, %	OR(95% CI)^a^	
BRM-1321	del/del	232(40.8)	60(55.6)	1.00(Reference)	101(44.3)	375(53.7)	1.00(Reference)	0.75
	ins/del	254(44.7)	39(36.1)	1.69 (1.06–2.69)	105(46.1)	279(40.0)	1.40(1.01–1.94)	
	ins/ins	82(14.4)	9(8.3)	2.37(1.08–5.37)	22(9.6)	44(6.3)	1.86(1.02–3.35)	
	*P*_trend_			0.004			0.007	
BRM-741	del/del	112(19.7)	23(21.3)	1.00(Reference)	46(20.2)	161(23.1)	1.00(Reference)	0.96
	ins/del	258(45.4)	49(45.4)	1.10(0.62–1.96)	114(50.0)	337(48.3)	1.17(0.78–1.77)	
	ins/ins	198(34.9)	36(33.3)	1.16(0.63–2.14)	68(29.8)	200(28.7)	1.19(0.76–1.87)	
	*P*_trend_			0.68			0.46	

a adjusted by age, sex, smoking status and drinking status.

### Association between BRM Haplotypes and HCC Risk

Linkage disequilibrium (LD) analyses revealed that BRM-741 and BRM-1321 polymorphisms were in a moderate LD (*D*′* = *0.68). Four inferred haplotypes were observed in the current samples. Results from the haplotype analysis were showed in Table 6. Consistent with results of the genotype analysis, the *BRM* haplotype containing both insertion risk allele of two polymorphisms (−1321 ins/−741 ins) was significantly associated with an enhanced risk of HCC, compared with the most common haplotype “−1321 del/−741 del ” (OR = 1.59, 95% CI = 1.33–1.90, *P*<0.0001).

**Table 6:** Association between *BRM* promoter haplotypes and risk of HCC^a^.

Population	Haplotype	Cases	n (%)	Controls	n (%)	OR (95% CI)	*P*
Panel I	−1321 del/−741 del	320	39.3	340	41.7	1.00 (reference)	
	−1321 ins/−741 ins	260	31.9	174	21.4	1.59(1.23–2.04)	**0.0002**
	−1321 del/−741 ins	202	24.7	260	31.8	0.83(0.65–1.06)	0.12
	−1321 ins/−741 del	34	4.1	42	5.1	0.86(0.52–1.42)	0.54
Panel II	−1321 del/−741 del	302	38.9	330	41.4	1.00 (reference)	
	−1321 ins/−741 ins	241	31.1	166	20.8	1.59(1.22–2.06)	**0.0003**
	−1321 del/−741 ins	201	25.9	258	32.5	0.85(0.66–1.09)	0.19
	−1321 ins/−741 del	32	4.1	42	5.3	0.83(0.50–1.39)	0.46
Overall	−1321 del/−741 del	622	39.1	670	41.6	1.00 (reference)	
	−1321 ins/−741 ins	501	31.5	340	21.1	1.59(1.33–1.90)	**<0.0001**
	−1321 del/−741 ins	403	25.3	518	32.1	0.84(0.70–1.00)	0.041
	−1321 ins/−741 del	66	4.1	84	5.2	0.85(0.59–1.21)	0.34

a Haplotype frequencies in cases and controls were compared using logistic regression.

### The Genotype-Phenotype Correlations between BRM Indel Polymorphisms and BRM Expression

**Figure 2:**
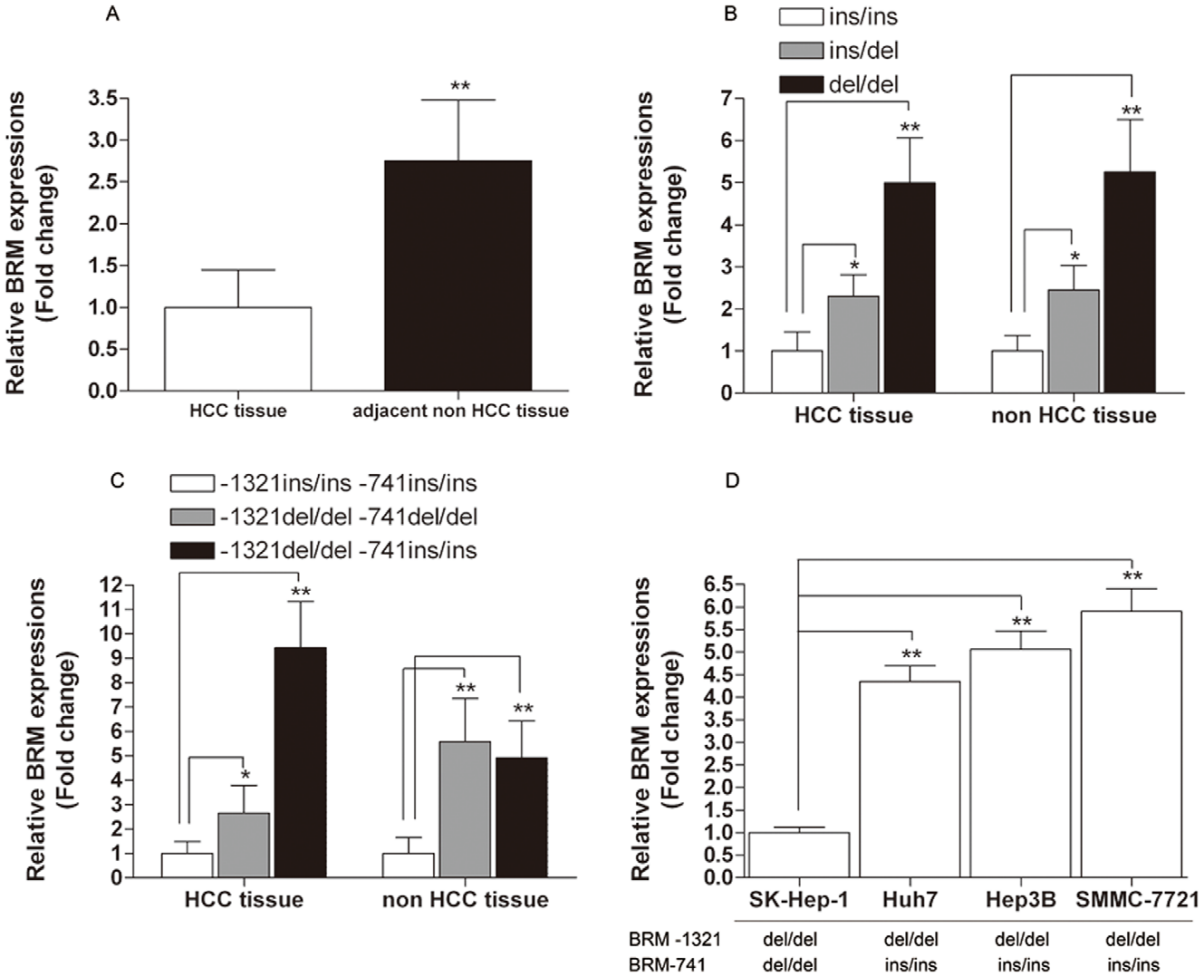
BRM expression in HCC tumor tissues *vs.* non-tumor tissues and its correlations between BRM-1321 indel polymorphism as well as corresponding haplotypes. (**A**) Relative BRM expression in HCC tumor tissues *vs.* non-tumor tissues (n = 72); (**B**) Relative BRM expression in three genotypic groups of BRM-1321 (−1321 ins/ins, n = 10, −1321 ins/del, n = 32, −1321 del/del, n = 30); (**C**) Relative BRM expression in different haplotype groups (haplotype: −1321 ins/ins-741 ins/ins, n = 6, haplotype: −1321 del/del-741 del/del, n = 11, haplotype: −1321 del/del-741 ins/ins, n = 4); (**D**) Relative BRM expression in hepatoma cell lines with different haplotypes. Data represented as mean ± SEM. *indicates *P*<0.01, **indicates *P*<0.001 compared within the same group (HCC tissue or non HCC tissue).

**Figure 3:**
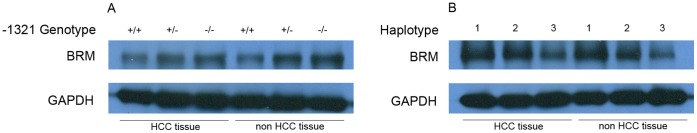
Western blot analysis of BRM expression in HCC tissue and adjacent non-tumor tissues. (A) BRM expression for different BRM-1321 genotypes. (B) BRM expression for different haplotypes. Haplotype #1: −1321 del/del-741 ins/ins, Haplotype #2: −1321 del/del-741 del/del, Haplotype #3: −1321 ins/ins-741 ins/ins.

To further explore the effect of BRM-1321 on the expression of BRM, we used three different genotypic HCC tissue samples as well as their adjacent non-tumor tissues to examine BRM expression. First, results of real-time PCR demonstrated that the expression level of BRM in adjacent non-tumor tissues was 2.75-fold higher than that of HCC tumor tissues (Figure 2A). Second, when we classified the tissue samples into three groups (ins/ins, ins/del and del/del) based on BRM-1321 genotype, significant differences were observed concerning BRM expression in both HCC tumor tissue and non-tumor tissues. Compared with ins/ins genotype, subjects carrying ins/del and del/del genotype had 2.30 and 4.99 fold higher BRM expression in HCC tissue samples, respectively. Similar trends were observed in adjacent non-tumor tissue samples (Figure 2B). Third, we further compared BRM expression levels of different haplotype groups according to the availability of appropriate genotype combinations of the indel polymorphisms. As shown in Figure 2C, compared with −1321 ins/ins-741 ins/ins haplotype, the BRM mRNA expression levels of −1321 del/del-741 del/del and −1321 del/del-741 ins/ins haplotypes were significantly increased from 2.65 to 9.43 fold. To validate our findings in HCC tissues, we further examined the genotype-phenotype correlations in four common hepatoma cell lines (Huh-7, Hep3B, sk-Hep-1 and SMMC-7721). Compared with sk-Hep-1 cell lines carrying −1321 del/del-741 del/del haplotype, the BRM mRNA expression levels of Huh-7, Hep3B and SMMC-7721 (−1321 del/del-741 ins/ins haplotype) were significantly increased (Figure 2D). Western blotting showed that BRM protein level of del/del and ins/del genotype carriers was higher than that with ins/ins genotypes (Figure 3A). Similarly, BRM protein expression of subject harboring −1321 del/del-741 ins/ins and −1321 del/del-741 del/del haplotype was higher than that with −1321 ins/ins-741 ins/ins haplotype (Figure 3B). Together, these data demonstrated that the genotypes of BRM-1321 and the corresponding haplotypes were significantly correlated with BRM expression *in vivo*, at both mRNA and protein levels.

## Discussion

To our knowledge, this is the first epidemiological study to assess the association between genetic variants of *BRM* gene and HCC risk. By analyzing two indel polymorphisms within the promoter region of BRM in two independent case control studies, we find that the genotypes of the BRM-1321 (rs3832613), not BRM-741 (rs34480940), can influence HCC incidence in Chinese populations. However, our findings suggest a significant interaction between BRM-741 and smoking behavior in HCC tumorgenesis. Functional assays reveal a significant genotype-phenotype correlation that the risk genotypes of BRM-1321 conferred lower BRM expression *in* *vivo*. These findings suggest that BRM promoter polymorphisms could regulate BRM expression and may serve as potential markers for genetic susceptibility to HCC.

BRM is absent or expressed at low levels in subsets of several types of tumor such as lung cancer and prostate cancer, pinpointing a central role for BRM loss in cancer development [9], [19]–[20]. Furthermore, BRM absence correlates with advanced stages of disease progression and poor prognosis [21], [22]. Similarly, inactivation of BRM can lead to an increased number of lung tumors in a mouse model [23]. Multiple lines of evidence have indicated that BRM may be regulated at both transcriptional and post- transcriptional levels [13], [24]. Our findings support the notion that the genetic variations within promoter region of *BRM* may be key functional elements in regulating expression of BRM. For example, BRM-1321 (rs3832613) may interrupt the bindings of specific transcription factors (i.e. myocyte enhancer factor-2) through which altering the BRM promoter activity, resulting in its misexpression. Numerous direct interactions have been identified between the SWI/SNF complex and well-known tumor-suppressor genes and oncogenes, such as RB and BRCA1 [25], [26]. Therefore, it is plausible that altered BRM expression may abrogate growth control by impairing RB-mediated cell cycle arrest. Meanwhile, BRM can promote the transcription of specific genes such as E-cadherin and CD44 by controlling recruitment and activation of methyltransferases or demethylases to their promoter sequences [27]. To this end, aberrant BRM expression conferred by promoter polymorphisms may also contribute to increased or repressed methylation of its target genes during tumor progression.

As a baseline, we first used real-time PCR to see if and how the BRM mRNA levels differed in HCC tumor tissues and adjacent non-tumor tissues. Consistent with previous findings [28], our results reveal that BRM expression in adjacent non-tumor tissues is significantly higher than that of HCC tumor tissues (Figure 2A). In deed, BRM has been also found to be differentially expressed between well-differentiated HCC and moderately-to-poorly differentiated HCC [29]. Moreover, we have shown that the ins/ins genotype of BRM-1321 is significantly associated with BRM expression in both HCC tumor tissues and adjacent non-tumor tissues, indicating this genotype-phenotype correlation is a ubiquitous phenomenon in human tissue.

It is worthy of note that we only observe a moderate LD (*D*′ = 0.68) of the two indels in the current study, which is different from that of previous results (*D'* = 0.83) [13]. Since LD may be influenced by population specific factors such as genetic drift or genomic region specific factors such as recombination rate [30], this discrepancy may due to population specific characteristic of these two indels. However, regulation of BRM may be mediated by multiple factors through different molecular mechanisms. For example, a miR-199a-dependent regulation of BRM has been suggested a potential feedback loop through EGR1 [31]. Thus, further studies will still be needed to investigate how BRM loss occurs and which major pathways affected by its loss, leading to the occurrence of HCC.

Tobacco smoking is one of the main known etiological factors of some cancers. Long-term tobacco smoking has been shown to contribute to carcinogenesis [32]. Smoking can significantly increase nuclear hypoxia-inducible factor (HIF)-1α expression, and SWI/SNF complex is required for HIF-1α mRNA [33]. The interaction between BRM-741 and smoking behavior in our study might be caused by alterations in catalytic efficiency between tobacco constituents and the polymorphic *BRM* gene. These findings provide a possible molecular explanation for the synergistic effect of smoking and genetic background on HCC development. However, details of the mechanism need to be verified by further well-designed experiments.

Finally, our case samples were only collected in two local comprehensive hospitals. Thus, we may not exclude any potential selection bias during sampling process. However, our case series can at least represent part of Chinese HCC patients. We should note that the current sample size is relative small especially for assessing three-way interactions. Therefore, further replication studies in ethnically different groups are necessary to fully establish the role of *BRM* polymorphisms in HCC and their relationships with other environmental factors implicated in HCC susceptibility.

In summary, our molecular epidemiological findings demonstrated a significant association of BRM-1321 with an increased risk of developing HCC in Chinese populations. Functional studies also provided new insights into the mechanisms that may explain the essential roles of BRM in modifying HCC susceptibility. Although these results confirmed BRM as a candidate gene for HCC in Chinese populations, the underlying molecular mechanism should be addressed clearly in future studies.
